# Delayed mGluR5 activation limits neuroinflammation and neurodegeneration after traumatic brain injury

**DOI:** 10.1186/1742-2094-9-43

**Published:** 2012-02-28

**Authors:** Kimberly R Byrnes, David J Loane, Bogdan A Stoica, Jiangyang Zhang, Alan I Faden

**Affiliations:** 1 Department of Neuroscience, Georgetown University Medical Center, Washington, DC, USA; 2 Department of Anatomy, Physiology and Genetics, Uniformed Services University of the Health Sciences, Bethesda, MD, USA; 3 Department of Anesthesiology & Center for Shock, Trauma and Anesthesiology Research (STAR), National Study Center for Trauma and EMS, University of Maryland School of Medicine, Baltimore, MD, USA; 4 Department of Radiology, Johns Hopkins University, Baltimore, MD, USA

**Keywords:** Traumatic brain injury, microglia, metabotropic glutamate receptor 5, delayed treatment, neuroprotection

## Abstract

**Background:**

Traumatic brain injury initiates biochemical processes that lead to secondary neurodegeneration. Imaging studies suggest that tissue loss may continue for months or years after traumatic brain injury in association with chronic microglial activation. Recently we found that metabotropic glutamate receptor 5 (mGluR5) activation by (*RS*)-2-chloro-5-hydroxyphenylglycine (CHPG) decreases microglial activation and release of associated pro-inflammatory factors *in vitro*, which is mediated in part through inhibition of reduced nicotinamide adenine dinucleotide phosphate (NADPH) oxidase. Here we examined whether delayed CHPG administration reduces chronic neuroinflammation and associated neurodegeneration after experimental traumatic brain injury in mice.

**Methods:**

One month after controlled cortical impact traumatic brain injury, C57Bl/6 mice were randomly assigned to treatment with single dose intracerebroventricular CHPG, vehicle or CHPG plus a selective mGluR5 antagonist, 3-((2-Methyl-4-thiazolyl)ethynyl)pyridine. Lesion volume, white matter tract integrity and neurological recovery were assessed over the following three months.

**Results:**

Traumatic brain injury resulted in mGluR5 expression in reactive microglia of the cortex and hippocampus at one month post-injury. Delayed CHPG treatment reduced expression of reactive microglia expressing NADPH oxidase subunits; decreased hippocampal neuronal loss; limited lesion progression, as measured by repeated T2-weighted magnetic resonance imaging (at one, two and three months) and white matter loss, as measured by high field *ex vivo *diffusion tensor imaging at four months; and significantly improved motor and cognitive recovery in comparison to the other treatment groups.

**Conclusion:**

Markedly delayed, single dose treatment with CHPG significantly improves functional recovery and limits lesion progression after experimental traumatic brain injury, likely in part through actions at mGluR5 receptors that modulate neuroinflammation.

## Background

Traumatic brain injury (TBI) causes cell death and neurological dysfunction through both direct physical disruption of tissue or pathways (primary injury), as well as delayed and potentially reversible molecular and cellular pathophysiological mechanisms (secondary injury) resulting in progressive white matter and grey matter damage [1]. Such delayed injury begins within seconds to minutes after the insult and may continue for days, weeks or potentially months to years [2]. These processes are characterized by neuronal cell death, as well as infiltration and activation of blood-borne immune cells, such as macrophages and lymphocytes, and activation of resident microglia [3].

After TBI, microglia become activated and undergo marked changes in cell morphology and behavior. Upon activation, microglia contract their processes and transform from a ramified to an ameboid cellular morphology, followed by proliferation and migration towards the site of injury [4]. Activated microglia can secrete a large number of factors, including cytokines, chemokines and other pro-inflammatory substances (for example, nitric oxide, prostaglandins and superoxide) that are toxic to neurons [5,6]. Microglial-mediated inflammation has been implicated as an important mechanism contributing to progressive neurodegeneration in multiple chronic neurological disorders [7]. Animal and clinical studies indicate that post-traumatic neuroinflammation persists for months to years after brain injury [8-11], and may contribute to chronic neurodegeneration and related significant neurological deficits [12-15].

We have previously identified a cluster of microglial associated genes and associated proteins that are chronically expressed after central nervous system (CNS) trauma [16], including membrane subunits of the reduced nicotinamide adenine dinucleotide phosphate (NADPH) oxidase enzyme. NADPH oxidase amplifies pro-inflammatory gene expression in activated microglia and promotes microglial-mediated neurotoxicity [17], and may be a key driving factor for the chronic progression of neurodegenerative disease [18]. More recently, we demonstrated that microglia in culture and in the rodent CNS express receptors for metabotropic glutamate receptor 5 (mGluR5). Activation of mGluR5 using the specific agonist (*RS*)-2-chloro-5-hydroxyphenylglycine (CHPG) inhibits microglial activation and the release of inflammatory factors, in part by inhibiting NADPH oxidase [19-21].

In the present studies we show that mGluR5 is chronically expressed in reactive microglia following controlled cortical impact (CCI) induced TBI in mice, and that single dose CHPG administration one month after TBI inhibits subsequent chronic post-injury inflammation and reduces the number of highly activated microglia that express NADPH oxidase. Such markedly delayed treatment also significantly reduces late tissue loss after TBI and improves long-term sensorimotor and cognitive recovery. As this effect is blocked by concurrent systemic administration of a selective mGluR5 receptor antagonist, the therapeutic actions of CHPG reflect actions at mGluR5 receptors.

## Methods

### Controlled cortical impact injury

All surgical procedures were carried out in accordance with protocols approved by Georgetown University Medical Center Institutional Animal Care and Use Committee. Our custom-designed CCI injury device [22] consists of a microprocessor-controlled pneumatic impactor with a 3.5 mm diameter tip. Male C57Bl/6 mice (20 to 25 g) were anesthetized with isoflurane evaporated in a gas mixture containing 70% N_2_O and 30% O_2 _and administered through a nose mask (induction at 4% and maintenance at 2%). Depth of anesthesia was assessed by monitoring respiration rate and pedal withdrawal reflexes. Mice were placed on a heated pad and their core body temperature was maintained at 37°C. The head was mounted in a stereotaxic frame, and the surgical site was clipped and cleaned with Nolvasan and ethanol scrubs. A 10-mm midline incision was made over the skull, the skin and fascia were reflected, and a 4-mm craniotomy was made on the central aspect of the left parietal bone. The impounder tip of the injury device was then extended to its full stroke distance (44 mm), positioned to the surface of the exposed dura, and reset to impact the cortical surface. Moderate-level injury was induced using an impactor velocity of 6 m/s and deformation depth of 2 mm as previously described [23]. After injury, the incision was closed with interrupted 6-0 silk sutures, anesthesia was terminated, and the animal was placed into a heated cage to maintain normal core temperature for 45 minutes post-injury. All animals were monitored carefully for at least 4 hours after surgery and then daily. Sham animals underwent the same procedure as injured mice except for the impact.

### Drug treatment

One month post-injury, mice received a single intracerebroventricular (icv) injection of CHPG or equal volume vehicle. A 10 mM solution (saline with 1% dimethyl sulfoxide) was injected into the left ventricle (coordinates from bregma = anteroposterior: -0.5, lateral: -1.0; ventral: -2.0) using a 30 gauge needle attached to a Hamilton syringe at a rate of 0.5 μL/min, with a final volume of 5 μL, or 50 nmols of CHPG. Fifteen minutes prior to CHPG administration, one group of mice received the selective mGluR5 antagonist 3-((2-Methyl-4-thiazolyl)ethynyl)pyridine (MTEP; 10 mg/kg in saline) by intraperitoneal injection. All other mice received an equal volume vehicle intraperitoneal injection. Dosages were based upon prior investigations in spinal cord injury and TBI models [20,24].

### *In vivo *magnetic resonance imaging

One, two and three months after CCI, brain lesion volume was assessed using T2-weighted magnetic resonance imaging (MRI) as previously described [23] (n = 6 per group). Briefly, anesthetized animals were placed in a heated plexiglass holder and a respiratory motion detector positioned over the thorax to facilitate respiratory gating. The plexiglass holder was then placed in the center of the 7 Tesla magnet bore (Bruker Medical Inc., Billerica, MA, USA) where a 72-mm proton-tuned birdcage coil was positioned. Field homogeneity across the brain was optimized and a sagittal scout image was acquired (rapid acquisition relaxation enhancement (RARE) pulse sequence image; field of vision, 4 × 4 cm; 128 × 128 resolution; repetition time (TR) to echo time (TE), 1,500/10 ms with a RARE factor of 8, making the effective TE 40 ms). Multi-slice, multi-echo T2-weighted images were acquired using the following parameters: field of vision, 3 × 3 cm; 256 × 256 resolution; TR to TE, 1,500/10 ms; RARE factor of 8; TE, 40 ms; 10 × slices; slice thickness, 0.75 mm. Lesion volume was quantified from the summation of areas of hyperintensity on each slice, multiplied by slice thickness, for both the ipsilateral and contralateral hemispheres. Contralateral volumes were subtracted from ipsilateral volumes to obtain TBI-induced lesion volumes.

### *Ex vivo *diffusion tensor imaging

*Ex vivo *diffusion tensor imaging (DTI) was performed four months post-injury on formalin-perfused brain (n = 5 per group). DTI imaging was performed with a 9.4 Tesla NMR spectrometer (Bruker Biospin, Billerica, MA, USA) equipped with a Micro2.5 gradient system (100 G/cm maximum gradient strength). We used our recently developed diffusion weighted gradient and spin echo sequence [25] with the following parameters: TE, 33 ms; TR, 900 ms; number of radio frequency pulses (Nrf) = 4; bandwidth, 100 kHz; and four signal averages. The imaging field of view and matrix size were 13.0 × 10.0 × 18.4 mm and 128 × 96 × 180 mm respectively, and the native resolution was approximately 100 × 100 × 100 μm^3^. The spectral data were apodized by a symmetric trapezoidal function with 10% ramp widths and zero-filled before Fourier transformation. For DTI, eight diffusion directions (*b*-value 1700 s/mm^2^), and two non diffusion-weighted images were acquired with δ = 3 ms, ∆ = 15 ms. The total imaging time was 11 hours. The signal-to-noise ratios in the corpus callosum measured in the non diffusion-weighted images were greater than 40 for all experiments.

### Image processing

For the *ex vivo *results, an average diffusion-weighted image (aDW) was obtained by averaging the six diffusion-weighted images. Signals from skull tissues in the aDW images were manually removed. The diffusion tensor was calculated using a log-linear fitting method [26,27]. The fractional anisotropy (FA), primary eigenvector (*v*_1_), parallel diffusivity (λ_║_, the primary eigenvalue) and perpendicular diffusivity (λ_┴_, the average of the secondary and tertiary eigenvalues) were calculated on a voxel-by-voxel basis from diffusion tensor using DTIStudio http://www.mristudio.org[28]. The *in vivo *and *ex vivo *aDW images were first rigidly aligned to our *ex vivo *MRI based atlases [29], respectively, using the six-parameter rigid registration function in the automated image registration package [30,31].

### Motor and cognitive function testing

Motor performance was assessed one, two and three months post-injury using the beam walk task, as previously described [22] (n = 7 (vehicle), 7 (CHPG), 4 (MTEP + CHPG)). Briefly, mice were trained to cross a narrow wooden beam 6 mm wide and 120 cm in length, which was suspended 300 cm above a 60-mm thick foam rubber pad. The number of foot-faults for the right hind limb was recorded over 50 steps and a basal level of competence at this task was established before surgery with an acceptance level of < 10 faults per 50 steps. Spatial learning and memory was assessed using an acquisition paradigm of the Morris water maze (MWM) test three and a half months post-injury as described [32] (n = 4 (naïve), 8 (vehicle), 8 (CHPG), 8 (MTEP + CHPG)). A white circular pool was divided into four quadrants using computer-based AnyMaze video tracking system (Stoelting Co., Wood Dale, IL, USA) and the platform was hidden in one quadrant (south-west) 35.56 cm from the side-wall. Spatial learning and memory performance was assessed by determining the latency (seconds) to locate the sub-merged hidden platform with a 90-second limit per trial. On the day after the MWM acquisition test reference spatial memory was assessed by a probe trial; the time spent (seconds) within a 60-second limit in the quadrant where the platform had been hidden during acquisition phase was recorded. A visual cue test was performed using a flagged platform placed on the platform in one of the quadrants (with a 90-second limit per trial) and latency (seconds) to locate the flagged platform was recorded. In addition, the Water maze search strategies employed by each animal during the acquisition trials of the MWM test were analyzed as previously described [33]. Briefly, three strategies were identified and categorized: spatial strategy was defined as swimming directly to the platform in no more than one loop or swimming directly to the correct target quadrant and searching; systematic strategy was defined as searching the interior portion of or entire tank without spatial bias, and searching incorrect target quadrant; and looping strategy was defined as circular swimming around the tank, swimming in tight circle, and/or swimming around the wall of tank.

### Unbiased stereological assessment of cortical microglia

At the indicated time points, mice were anesthetized (100 mg/kg sodium pentobarbital, intraperitoneal injection) and transcardially perfused with 100 mL of 0.9% saline followed by 300 mL of 4% paraformaldehyde (10% buffered formalin solution, Fisher Scientific, Pittsburg, PA, USA) (n = 5 per group). The brain was removed and post-fixed in 4% paraformaldehyde overnight and cryoprotected in 30% sucrose. Coronal sections were cut (three × 60 μm followed by three × 20 μm sections) and serially collected throughout the injured brain, starting at +1.78 mm from the bregma. Sections were mounted onto glass slides for immunohistochemical analysis. Microglia were immunostained with anti-ionized calcium binding adaptor molecule 1 (Iba-1) (1:1000; Wako Chemicals, Richmond, VA, USA) for 1 hour, washed in PBS and incubated with biotinylated anti-rabbit immunoglobulin G antibody (Vector Laboratories, Burlingame, CA, USA) for 2 hours at room temperature. Sections were placed in avidin-biotin-horseradish peroxidase solution, diluted according to the manufacturer's instructions for 1 hour (Vectastain elite ABC kit, Vector Laboratories) and then reacted with 3,3'- diaminobenzidine (Vector Laboratories) for color development. Sections were counterstained with cresyl violet (FD NeuroTechnologies, Baltimore, MD, USA), dehydrated and mounted for analysis.

Stereoinvestigator software (MBF Biosciences, Williston, VT, USA) was used to count the number of cortical microglia in each of the three microglial morphological phenotypes (ramified, hypertrophic and bushy) using the optical fractionator method of unbiased stereology. The sampled region was the perilesional ipsilateral cortex between -1.22 mm and -2.54 mm from the bregma. Every fourth 60-μm section was analyzed beginning from a random start point. Sections were analyzed using a Leica DM4000B microscope (Leica Microsystems, Exton, PA, USA). The optical dissector had a size of 50 × 50 μm in the x and y-axis with a height of 10 μm and guard zone of 4 μm from the top of the section. Dissectors were positioned every 150 μm in the x and y-axis. Microglial phenotypic classification was based on the length and thickness of the projections, the number of branches and the size of the cell body as previously described [34]. Neurolucida software (MBF Biosciences) was used to trace and quantify the size of microglial cell bodies and dendrites at different stages of activation following injury. Microglia were outlined using the live image setting so that the width of the dendrites could be traced while focusing on the section. The cell body was outlined using the contour tool followed by the tracing of the individual dendrites, using the dendrite line tool. A quick measure tool quantified the average thickness of each microglial branch. Neurolucida explorer software was used to determine the cell body area and volume, and dendrite length and branching number. Ramified microglia possessed long thin processes (> 650 μm in length), had a small cell body volume (< 10 μm^3^) and many branches (20 to 30). Hypertrophic microglia possessed medium length processes (300 to 550 μm in length), had larger cell body volumes (50 to 75 μm^3^) and many branches (20 to 30). Bushy microglia possessed short thick processes (< 200 μm in length), had a larger cell body volume (80 to 100 μm^3^) and very few branches (< 10). The volume of the region of interest was measured using a Cavalieri estimator method with a grid spacing of 100 μm. The estimated number of microglia in each phenotypic class was divided by the volume of the region of interest to obtain the cellular density expressed in cells per cubic millimeter.

### Unbiased stereological counting of surviving hippocampal neurons

The stereoinvestigator software was used to count the total number of surviving neurons in the *Cornu Ammonis *(CA) 1, CA3 and dentate gyrus subfields of the hippocampus using the optical fractionator method of unbiased stereology. The sampled region for each hippocampal subfield was demarcated in the injured hemisphere and cresyl violet neuronal cell bodies were counted. The volume of the hippocampal subfield was measured using a Cavalieri estimator method. The estimated number of surviving neurons in each field was divided by the volume of the region of interest to obtain the cellular density expressed in cells per cubic millimeter.

### Immunohistochemistry

Immunohistochemistry was performed on 20-μm sections and standard immunostaining techniques were employed. The following primary antibodies were used: rabbit anti-Iba-1 (1:500, cat. no. 019-19741, Wako Chemicals), mouse anti-mGluR5, clone 464823 (1:100; cat. no. MAB45141, R&D Systems, Minneapolis, MN, USA), rat anti-ED1 (1:200, cat. no. MCA1957G, AbD Serotec, Raleigh, NC, USA), and mouse anti-gp91^phox ^(1:200, cat. no. 611415, BD Transduction Inc., Franklin Lakes, NJ, USA). Counterstaining was performed with 4', 6-diamidino-2-phenylindole (1 μg/mL; Sigma-Aldrich, St. Louis, MO, USA). Fluorescence microscopy was performed using a LEICA (TCS SP5 II) confocal microscope system (Leica Microsystems).

### Statistical analysis

Lesion volume, functional data and unbiased stereological analysis were performed by an investigator blinded to treatment group. Quantitative data were expressed as mean ± standard errors of the mean (SEM). Functional data for beam walk and acquisition phase of MWM were analyzed by repeated measures (trial and time) one-way (groups) analysis of variance (ANOVA) to determine the interactions of post-injury trial and groups, followed by post-hoc adjustments using Student-Newman-Keuls test. Search strategy analysis was analyzed using a chi-square analysis. The T2-weighted MRI analysis was analyzed by two-way ANOVA, followed by post-hoc adjustments using Bonferroni *t*-test. Remaining data were analyzed using Mann-Whitney *U *test, Student's *t *test or one-way ANOVA, where appropriate. The functional data was analyzed using SigmaPlot 12 (Systat Software, San Jose, CA, USA). All other statistical tests were performed using the GraphPad Prism Program, Version 3.02 for Windows (GraphPad Software, San Diego, CA, USA). *P *< 0.05 was considered statistically significant.

## Results

### mGluR5 is expressed in chronically activated microglia after traumatic brain injury

After TBI, microglia undergo dramatic changes in cell morphology and behavior, transforming from a resting status that have a ramified cellular morphology to highly reactive cells that have a dense and enlarged amoeboid-like cell morphology (bushy or hypertrophic) and up-regulate cell surface antigens such as CD68 or ED1. We have recently demonstrated that microglial activation is associated with progressive neurodegeneration up to 21 days post-TBI [35], and that mGluR5 is expressed in cultured microglia [19,21] and co-localizes with microglia in a rodent model of spinal cord injury [20].

**Figure 1:**
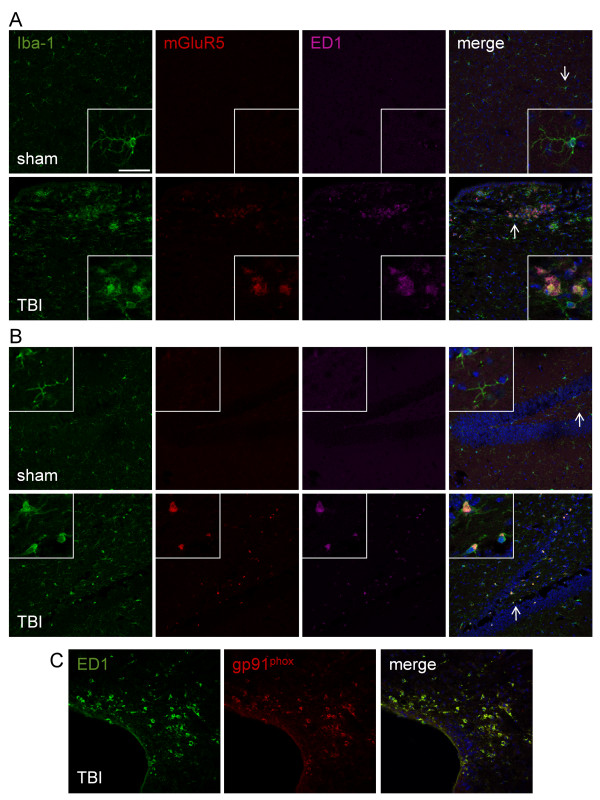
**mGluR5 is expressed in chronically activated microglia at one month post TBI. (A and B) **mGluR5 expression was evaluated in the cortex (A) and hippocampus (B) of sham or TBI brains at one month post-injury. Iba-1 (green) and ED1 (magenta) labeled activated microglia. mGluR5 expression (red) was undetectable in resting microglia that displayed a ramified cellular morphology, but was strongly up-regulated in highly reactive microglia that displayed a hypertrophic or bushy cellular morphology and co-expressed ED1 (merged). **(C) **The membrane bound component of the NADPH oxidase enzyme, gp91^phox ^(red), co-localized (merged) with ED1-positive reactive microglial (green) at one month post-TBI. Bar = 25 μm.

In order to evaluate mGluR5 expression levels in chronically activated microglia, C57Bl/6 mice underwent moderate-level CCI TBI or sham surgery and samples were collected one month after TBI for immunohistochemical analysis. mGluR5 expression was undetectable in resting microglia that displayed a ramified cellular morphology in the cortex and hippocampus of sham-injured samples (Figure 1A, B). In contrast, mGluR5 expression was up-regulated in highly reactive microglia that displayed a hypertrophic or bushy cellular morphology and co-expressed ED1 cell-surface antigens in the cortex and hippocampus of one-month TBI samples. Notably, the membrane bound component of the NADPH oxidase enzyme, gp91^phox^, co-localized with ED1-positive reactive microglial in the injured cortex at one month post-injury (Figure 1C), indicating chronic expression of NADPH oxidase in microglia following TBI.

### Delayed administration of CHPG reduces lesion volume and increases white matter integrity after traumatic brain injury

**Figure 2:**
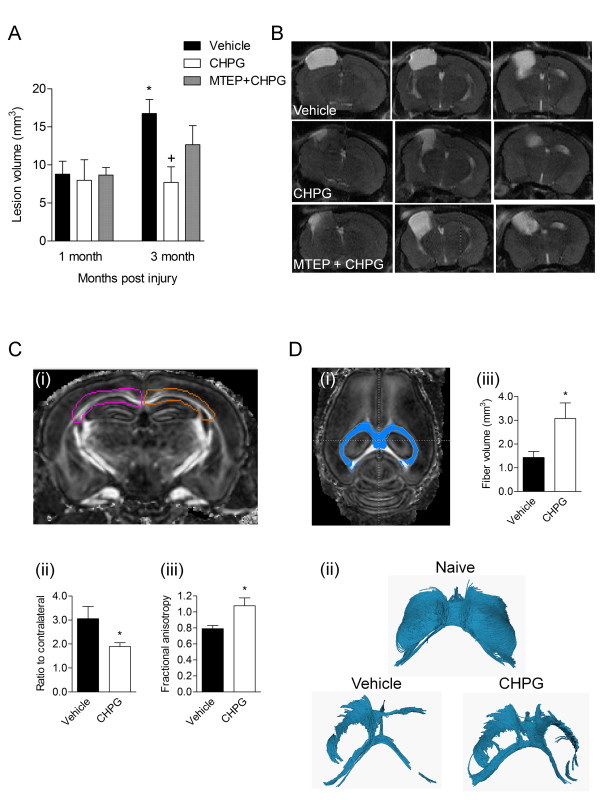
**Delayed treatment with the mGluR5 agonist, CHPG, reduces lesion volume and increases white matter integrity after TBI. (A) **Lesion volume was assessed using T2-weighted MRI in TBI mice. One month post-injury, all TBI mice had significant and comparable lesions volumes and were subsequently randomized and treated with vehicle, CHPG or MTEP + CHPG. TBI-induced lesion volumes significantly increased over three months in the vehicle-treated TBI group (*P < 0.05 versus one month), whereas the lesions in the CHPG-treated TBI group did not expand and were significantly reduced at three months post-injury when compared to the vehicle-treated TBI group (+*P *< 0.05 versus vehicle). TBI mice that received MTEP co-administration with CHPG had lesion volumes that were not significantly different compared to vehicle-treated TBI levels (n = 6 per group). **(B) **Representative MRI images show hyperintense lesion areas from the lesion epicenter and rostral/caudal regions at three months post-injury for each treatment group. **(C) ***Ex vivo *DTI was performed at four months post-injury. White matter tracts of interest were outlined in either color or FA images (Ci) on both the ipsilateral (pink) and contralateral (brown) sides. The ratio of the ipsilateral mean diffusivity (Cii) and FA (Ciii) to contralateral measurements demonstrate that CHPG treatment significantly reduced mean diffusivity and increased FA (*P < 0.05, n = 5). **(D) **White matter tracts were outlined (Di) to determine fiber volume (Diii). Three-dimensional visual representations are presented in (Dii). A naïve sample demonstrates the standard volume of white matter tracts in uninjured tissue. TBI resulted in a marked reduction in white matter volume. Fiber volume was significantly increased in CHPG-treated TBI tissue when compared to vehicle-treated TBI tissue (*P < 0.05, n = 5). Statistical analysis was by two-way ANOVA with Bonferroni *t*-test post-hoc corrections in (A), Mann-Whitney *U *test in (Cii), and Student's *t*-test in (Ciii) and (Diii). Bars represent mean ± SEM.

Given that microglia are chronically activated and express NADPH oxidase after TBI, we hypothesized that markedly delayed inhibition of selected inflammatory pathways may attenuate microglial-mediated inflammation and reduce progressive neurodegeneration after brain trauma. Previously we demonstrated that the selective mGluR5 agonist, CHPG, inhibits microglial activation and the release of associated inflammatory factors *in vitro*, in part mediated by inhibition of NADPH oxidase [19,21]. Therefore, at one month post-injury, TBI mice underwent T2-weighted MRI to assess lesion volume and were subsequently randomized prior to treatment to insure that all groups had comparable lesion volumes (at one month post-injury: vehicle = 8.8 ± 1.7 mm^3^, CHPG = 8.0 ± 2.7 mm^3^, MTEP + CHPG = 8.7 ± 1.0 mm^3^, mean ± SEM). After randomization, TBI mice were administered one of three treatment regimens at one month post-TBI: a single dose icv injection of CHPG; equal-volume vehicle (saline); or intraperitoneal injection of MTEP (mGluR5 antagonist) prior to icv CHPG administration. In vehicle-treated TBI mice, the lesion volume expanded significantly between one and three months post-injury (Figure 2A; *P *= 0.016), increasing to 190% of one-month (pre-treatment) values, indicating progressive lesion expansion over time. In contrast, CHPG treatment arrested the expansion of the lesion over time with lesion volumes at three months post-injury not significantly different from one-month (pre-treatment) values. Moreover, at three months post-injury, CHPG-treated TBI mice had significantly reduced lesion volumes when compared to vehicle-treated TBI mice (Figure 2A; vehicle-treated TBI = 16.8 ± 1.9 mm^3 ^versus CHPG-treated TBI 7.7 ± 2.0 mm^3^, *P *= 0.010). In the negative control group, administration of the mGluR5 antagonist MTEP 15 minutes prior to CHPG treatment prevented the beneficial effects of CHPG, with lesion volumes of MTEP + CHPG-treated mice not significantly different to vehicle-treated mice (Figure 2A; MTEP + CHPG-treated TBI = 12.66 ± 2.5 mm^3^). Similar trends were detected two months post-injury but did not reach statistical significance (vehicle-treated TBI = 10.02 ± 1.9 mm^3^, CHPG-treated TBI 7.6 ± 2.4 mm^3^, MTEP + CHPG-treated TBI = 9.128 ± 1.3 mm^3^). Representative MRI images from each treatment group at three months post-injury are presented in Figure 2B.

*Ex vivo *DTI using a 9.4 Tesla NMR spectrometer imaging system was performed four months post-injury to analyze white matter tract integrity in TBI mice. TBI increased mean diffusivity and decreased FA in vehicle-treated TBI mice, whereas delayed CHPG treatment significantly reduced mean diffusivity (Figure 2Cii; *P *= 0.032) and increased FA (Figure 2Ciii; *P *= 0.048) in CHPG-treated TBI mice, suggesting significant sparing of white matter tracts at four months post-TBI. Further analysis revealed that CHPG treatment significantly increased the external capsule fiber volume after TBI (Figure 2D; vehicle-treated TBI = 1.4 ± 0.3 mm^3 ^versus CHPG-treated TBI = 3.1 ± 0.7 mm^3^, mean ± SEM, *P = *0.024). These data demonstrate that delayed treatment with the mGluR5 agonist CHPG at one month post-TBI resulted in improved structural integrity and preserved white mater tracts at four months post-TBI.

### Delayed treatment with CHPG improves functional recovery after traumatic brain injury

**Figure 3:**
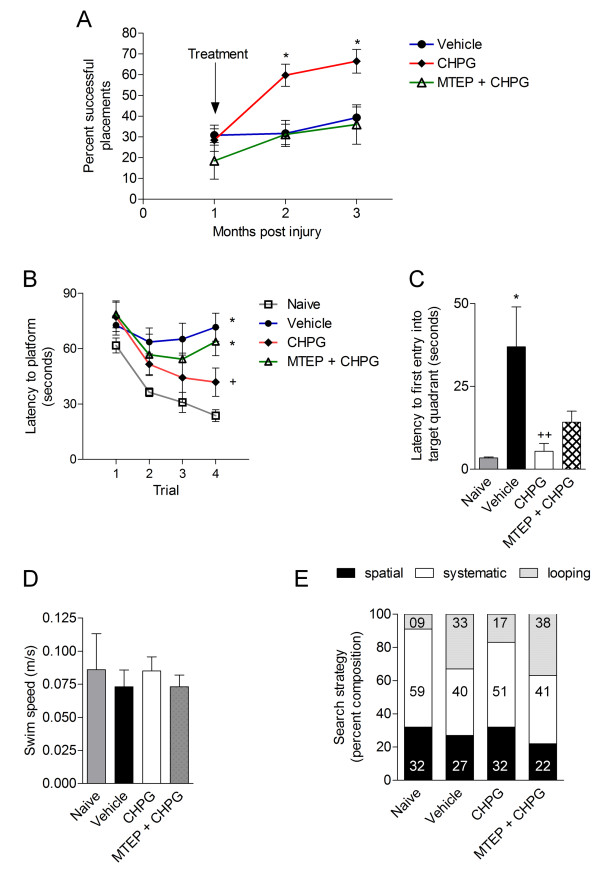
**Delayed treatment with CHPG improves functional recovery after TBI. (A) **Motor coordination was assessed using a beam walk test in TBI mice at one, two and three months post-injury. All injured mice performed poorly on this task prior to treatment, with no significant differences between treatment groups. At two and three months post-injury, CHPG-treated TBI mice showed a significant increase in the number of correct hind limb placements when compared to vehicle-treated mice (*P < 0.05 versus vehicle). Co-administration of MTEP with CHPG blocked CHPG-mediated improvements such that this group performed similar to vehicle-treated TBI mice (n = 7 (vehicle), 7 (CHPG), 4 (MTEP + CHPG)). **(B) **Spatial learning and memory was assessed using a one-day MWM protocol performed three and a half months post-TBI. The latency to find the submerged hidden platform in trial 4 was significantly increased in vehicle- and MTEP + CHPG-treated TBI mice when compared to naïve controls (*P <*0.05 versus naïve). CHPG treatment significantly reduced the latency when compared to vehicle-treated TBI mice (*P *< 0.05 versus vehicle) (n = 4 (naïve), 8 (vehicle), 8 (CHPG), 8 (MTEP + CHPG)). **(C) **One day after MWM mice performed a probe trial in which the platform was removed and the time to enter the correct quadrant where the platform had been was measured. Vehicle-treated TBI mice had significantly increased latency to first entry into target quadrant compared to naïve controls (*P *< 0.05 versus naïve). CHPG-treated TBI mice had reduced retention memory deficits and had significantly reduced latency to first entry (*P *< 0.01 versus vehicle). **(D) **There were no significant differences in swim speeds between any of the treatment groups throughout the trials. **(E) **The escape strategy in the MWM test was analyzed, and the percent composition for each strategy (spatial, systematic and looping) demonstrated that vehicle- and MTEP + CHPG-treated TBI mice had diminished spatial and systematic strategies and increased looping strategies compared to naïve or CHPG-treated TBI mice. Statistical analyses were by repeated measures one-way ANOVA with Student-Newman-Keuls post-hoc corrections in (A) and (B), one-way ANOVA with Student-Newman-Keuls post-hoc corrections in (C) chi-square analysis in (E). Bars represent mean ± SEM.

In order to evaluate the long-term therapeutic benefit of delayed administration of the mGluR5 agonist on TBI-induced neurological impairments, we performed motor and cognitive functional testing through the four months post-injury. Functional assessment of fine motor co-ordination was performed at one, two and three months after TBI using a beam walk test. Prior to treatment at one month post-TBI, all groups of mice had comparable motor function deficits (Figure 3A). Vehicle-treated TBI mice failed to improve motor performance at two or three months post-injury; in contrast, CHPG-treated TBI mice showed significant motor function recovery at both time points in the beam walk task (Figure 3A; successful foot placements at three months post-injury: vehicle-treated TBI = 39.3 ± 5.1% versus CHPG-treated TBI = 66.5 ± 5.7%, mean ± SEM, *P *< 0.05). Furthermore, MTEP pre-treatment blocked this effect, with MTEP + CHPG-treated TBI mice performing identically to vehicle-treated mice throughout.

A one-day MWM test [32] was performed at three and a half months post-TBI to assess spatial learning and memory. The interaction of 'post-injury trial x groups' (F(9,92) = 0.663, *P *= 0.74) was not statistically significant. However, the factors of 'post-injury trial' (F(3,92) = 5.032, *P *= 0.003) and 'groups' (F(3,92) = 5.731, *P *= 0.001) were found to be significant. Vehicle- and MTEP + CHPG-treated TBI mice performed poorly in this cognitive task, showing considerably increased latency to find the hidden platform in trial 4 when compared with uninjured, naïve mice (Figure 3B; *P *= 0.008 (vehicle), *P *= 0.016 (MTEP + CHPG), versus naïve). CHPG-treated TBI mice showed improvements in cognitive performance with significantly reduced latency to find the submerged platform in trial 4 when compared with vehicle-treated TBI mice (*P *= 0.024, versus vehicle). Reference memory was assessed using a probe trial on the day following the MWM acquisition trials and time for first entry into the target quadrant in which the platform was originally placed was recorded. CHPG-treated TBI mice had significantly reduced latency into the target quadrant when compared to vehicle-treated TBI mice (Figure 3C; *P *< 0.01), indicating reduced retention memory deficits in the probe trial. Swim speeds did not differ across groups (Figure 3D) and all mice performed well in the visual cue test (data not shown).

In order to assess the search strategies utilized by mice to find the hidden platform, the swimming pattern for each mouse was analyzed and assigned a search strategy (spatial, systematic or looping) [33]. The comparison of search strategies between groups was performed by chi-square analysis. Vehicle-treated and MTEP + CHPG-treated TBI mice displayed diminished spatial and systematic search strategies and increased looping strategy compared to naïve or CHPG-treated TBI mice (Figure 3E; *P *< 0.05, ^*χ*2 ^= 16.35). Taken together, the behavioral data indicate that delayed CHPG administration improves motor and cognitive recovery following TBI through an mGluR5-mediated mechanism.

### Delayed treatment with CHPG reduces the number of activated microglial in the injured cortex and attenuates hippocampal neurodegeneration at four months post-injury

**Figure 4:**
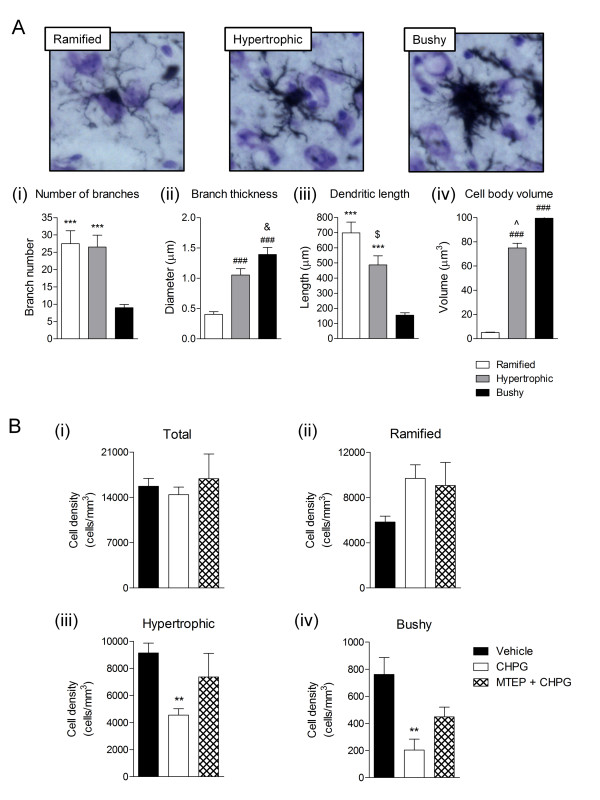
**CHPG treatment reduces the number of hypertrophic and bushy microglia in the injured cortex at four months post-injury. (A) **Representative Iba-1 immunohistochemical images displaying each microglial activation phenotype. Microglia was reconstructed using Neurolucida software and classified into ramified, hypertrophic and bushy activation phenotypes based on cellular morphological features. Analysis of the difference between the three activation phenotypes indicated marked changes in dendritic length (Ai), branch thickness (Aii), number of branches (Aiii) and cell body volume (Aiv). ***P < 0.001 versus bushy, ^###^*P *< 0.001 versus ramified, ^&^*P *< 0.05 versus hypertrophic, ^$^*P *< 0.05 versus ramified, ^*P *< 0.05 versus bushy. **(B) **Unbiased stereological quantification of microglial cell number and activation phenotype in each treatment group four months post-injury. There was no significant difference in the total number of microglia (Bi) in the injured cortex four months post-injury. CHPG-treated TBI tissue had significantly reduced numbers of hypertrophic (Biii) and bushy (Biv) microglia when compared to vehicle-treated TBI tissue, and increased numbers of ramified microglia (Bii). **P <0.01 versus vehicle,(n = 5/group). MTEP + CHPG-treated TBI tissue showed similar microglial phenotype profiles as the vehicle-treated TBI tissue, with fewer ramified microglia and more hypertrophic and bushy phenotypes. Statistical analyses were by one-way ANOVA with Student-Newman-Keuls post-hoc corrections in (A) and (B). Bars represent mean ± SEM.

Following completion of the behavioral and *in vivo *MRI studies, TBI mice were euthanized, their brains fixed and coronal sections cut for histological studies. Iba-1 immunohistochemistry was performed to label microglia in the injured brain. Based on cell morphological features, microglia can be classified into three categories corresponding to increasing activation status: ramified (resting), hypertrophic and bushy [34,36,37]. Ramified microglia have small cell bodies and thin, long and highly branched processes (Figure 4A). In contrast, hypertrophic microglia have larger cell bodies, with thicker, shorter and highly branched processes, whereas bushy microglia have multiple short processes that form thick bundles around enlarged cell bodies (Figure 4A). CNS injuries, such as stroke and TBI, cause transformation of resting ramified microglia into more active phenotypes, such as hypertrophic and bushy forms [34,35]. We performed an unbiased stereological assessment of microglial cell number and activation phenotype in the injured cortex of each group of mice at four months post-TBI. Our data showed that despite an equal number of total microglia in each treatment group there was a significantly reduced number of reactive hypertrophic and bushy microglia in the cortex of CHPG-treated TBI mice when compared to vehicle-treated TBI mice (Figure 4B; *P *< 0.01).

**Figure 5:**
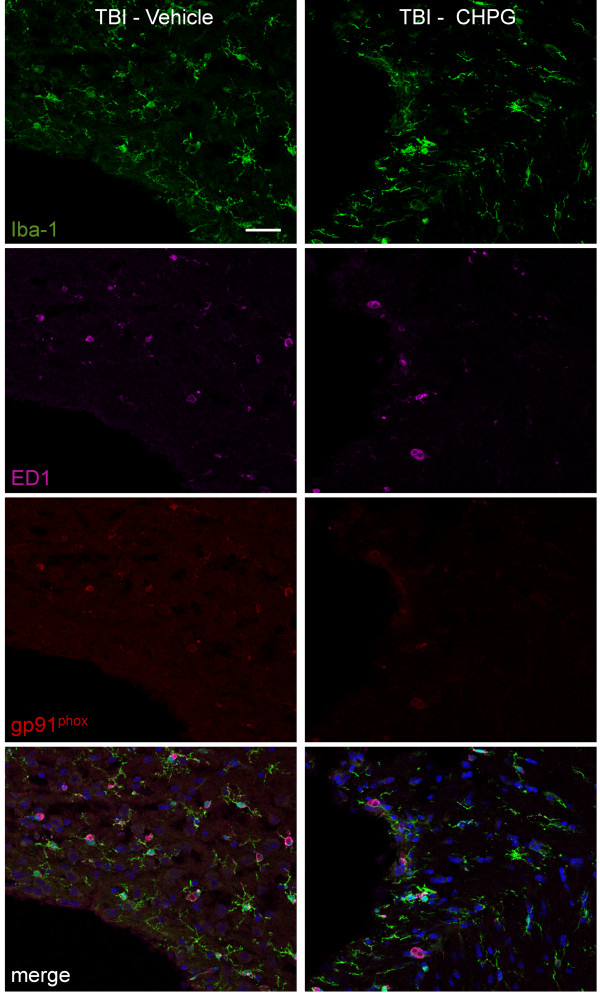
**CHPG treatment attenuates NADPH oxidase expression in reactive microglia at four months post-injury**. The NADPH oxidase subunit, gp91^phox ^(red), co-localized (merged) with EDI-positive reactive microglial (magenta) that displayed a hypertrophic or bushy cell morphology (Iba-1; green) in the injured cortex of vehicle-treated TBI samples four months post-TBI. CHPG-treated TBI samples has reduced gp91^phox^-positive reactive microglia at this time point. Bar = 25 μm.

We then performed triple immunofluorescence staining for reactive microglia (Iba-1/ED1-positive) that expressed NADPH oxidase (gp91^phox^-positive) in the four month vehicle-treated and CHPG-treated TBI samples (Figure 5). gp91^phox ^was highly expressed in ED1-positive microglia that displayed a hypertrophic or bushy cellular morphology in the cortex adjacent to the lesion in vehicle-treated TBI samples. Notably, there were fewer reactive microglia (ED1-positive) in the CHPG-treated TBI samples and gp91^phox ^expression was also markedly reduced in these cells. These data demonstrate that delayed CHPG treatment selectively reduced the activation of the highly reactive hypertrophic and bushy microglia at four months post-TBI and reduced the expression of NADPH oxidase in these cells.

**Figure 6:**
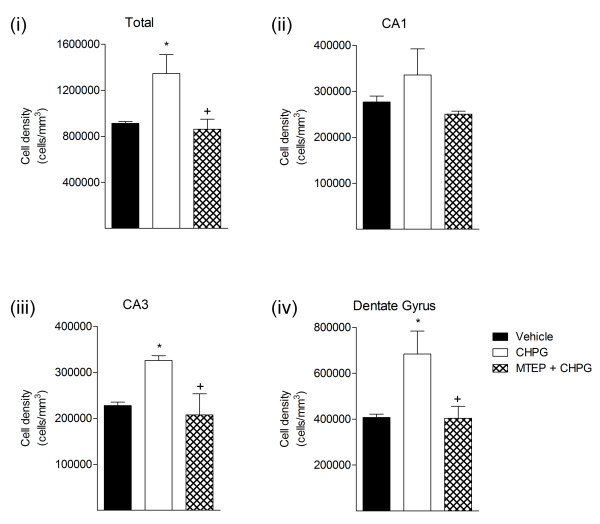
**Unbiased stereological quantification of hippocampal neurons at four months post-injury**. CHPG-treatment resulted in significantly reduced TBI-induced total neuron (Ai) loss in the hippocampus when compared to vehicle-treated samples. In addition, CHPG-treatment significantly spared neurons in the CA3 (Aiii) and dentate gyrus (Aiv) sub-subfields of the hippocampus (*P < 0.05, ***P < 0.001 versus vehicle; n = 3). In contrast, co-administration of MTEP with CHPG reversed the neuroprotective effects of CHPG-treatment in each hippocampal sub-field (+*P *< 0.05 versus CHPG). Statistical analysis was by one-way ANOVA with Student-Newman-Keuls post-hoc corrections. Bars represent mean ± SEM.

Finally, we performed stereological assessment of surviving neurons in the CA1, CA3 and dentate gyrus subfields of the hippocampus at four months post-TBI to evaluate post-traumatic neuronal loss. CHPG treatment significantly reduced neuronal cell loss in the hippocampus four months post-injury (Figure 6), with greater numbers of surviving neurons when compared to vehicle-treated and MTEP + CHPG-treated TBI groups. Moreover, CHPG-treatment resulted in significantly increase neuronal survival in the CA3 and dentate gyrus subfields of the hippocampus when compared to the vehicle-treated TBI group (Figure 5; CA3: 227,897 ± 7,363 (vehicle) versus 326,085 ± 10,203 (CHPG) counts/mm^3^, *P *< 0.001; dentate gyrus: 407,322 ± 14,244 (vehicle) versus 683,886 ± 100,182 (CHPG) counts/mm^3^, *P *< 0.05). The neuroprotective effects of CHPG treatment were abolished by co-administration of the mGluR5 antagonist, MTEP (Figure 6; CA3: 207,661 ± 45,864 (MTEP + CHPG) counts/mm^3^; dentate gyrus: 403,799 ± 52,466 (MTEP + CHPG) counts/mm^3^, both *P *< 0.05 versus CHPG-treated TBI group).

## Discussion

Taken together, our data demonstrate chronic microglial activation after experimental TBI, which may contribute to the observed progressive neurodegeneration and tissue loss that is associated with functional impairments. Furthermore, they demonstrate that the temporal window for neuroprotective intervention after TBI is significantly longer than generally believed. These observations are consistent with prior experimental work demonstrating progressive cortical damage and increased NFκB activation in macrophages and/or microglia up to one year after trauma [12,38,39]. Prolonged microglial activation has been demonstrated many months after TBI in humans [9] and increased microglial activation has also been observed up to four years after TBI in human post-mortem tissue [11].

We previously reported that CHPG treatment, acting through mGluR5, reduces microglial activation and the associated release of free radicals and pro-inflammatory cytokines in microglial cell culture models (both primary cultures and a mouse microglial cell line) after stimulation with the classical activators lipopolysaccharide or interferon-γ [19,21]. CHPG treatment also abolished the neurotoxic potential of activated microglia and reduced NADPH oxidase activity in such *in vitro *models. These effects of CHPG were blocked by knockout of the mGluR5 receptor or by addition of the selective mGluR5 antagonist MTEP, and reduced by co-incubation with siRNAs directed against either of the two membrane subunits of NADPH oxidase [19]. The current study supports these findings, as MTEP administration prior to CHPG treatment blocked its protective actions.

Examining the injured cortex one month after TBI, we demonstrated that mGluR5 expression was up-regulated in activated microglia that co-expressed the phagocytic marker, ED1, and exhibited a hypertrophic or bushy cellular phenotype. The observed post-traumatic expression of mGluR5 in activated microglia is consistent with previous *in vivo *studies demonstrating microglial mGluR5 expression at the lesion site in spinal cord injury and excitotoxic brain injury models [20,40]. In response to trauma, there is both microglial proliferation and activation, along with migration to the site of injury [41,42]. In contrast to the ramified appearance of resting microglia, activated microglia undergo substantial morphological changes, with reduction and thickening of processes leading to a hypertrophic or bushy appearance [34]. Although microglia are believed to have both neurotoxic and neuroprotective properties [3,5,43], considerable experimental data suggest that post-traumatic inflammation can contribute to delayed cell and tissue loss [35,44,45]. Indeed, microglial activation and release of associated inflammatory factors has been proposed as an important contributing factor for many acute and chronic neurodegenerative disorders [7,46].

Here we demonstrate that delayed CHPG administration significantly reduced the number of microglia showing the reactive bushy or hypertrophic phenotypes associated with a pro-inflammatory and potentially neurotoxic state [47]. Moreover, activated microglia expressed the NADPH oxidase sub-unit, gp91^phox ^four months post-injury, suggesting the chronic expression of NADPH oxidase in these cells; these data are consistent with the chronic up-regulation of expression of NADPH oxidase sub-units in a microglial-associated gene cluster six months after spinal cord injury [16]. Activated microglia cause neuronal cell death in culture through mechanisms that involves NADPH oxidase activation, and this process is inhibited by CHPG treatment [19,21]. Activated microglia also have been implicated in chronic functional deficits after TBI in humans [9]. Recently, inhibition of NADPH oxidase has been shown to reduce microglial activation in the post-ischemic brain [48]. Notably, single dose CHPG administration one month after TBI reduced the expression of NADPH oxidase in reactive microglia at four months post-injury. Together, these data suggest a positive activation feedback loop for neuroinflammation that contributes to delayed neurodegeneration and related functional deficits. Interruption of this positive feedback loop may explain why even a single injection of CHPG administered one month post-injury inhibits chronic neuroinflammation and limits functional loss in our model.

mGluR5 is also expressed in other cell types of the CNS, such as neurons, astrocytes and oligodendrocytes [49,50]. In addition to their anti-inflammatory effects, group I mGluR agonists also reduce neuronal apoptosis [51] as well as oligodendrocyte cell death [52]. Although it is possible that anti-apoptotic effects of CHPG on neurons or oligodendrocytes may have contributed to the recovery observed in the current study, such apoptotic processes so late after injury are likely limited and, given the half life of the compound, modulation of such events would probably contribute minimally at best to the improved outcome observed. In order to clarify the mechanism underlying mGluR5-mediated neuroprotection after TBI, future studies will require cell specific mGluR5 knockout in neurons, astrocytes, oligodendrocytes and microglia.

It is widely accepted that the therapeutic window for limiting post-traumatic neurodegeneration after acute brain injury is limited [53]. Indeed, most experimental treatment studies for TBI have focused on the first hours after injury [1]. With the recognition that more delayed apoptotic mechanisms may contribute to injury [54,55], the potential therapeutic window has been expanded to perhaps 24 to 72 hours. Although it has been shown experimentally and, more recently, clinically that tissue loss after TBI may progress for months or longer [12,14,15,56,57], there have been few attempts to pharmacologically modify such markedly delayed neurodegeneration. Here we demonstrate that single dose treatment one month after trauma significantly reduced both histological changes and behavioral dysfunction over a subsequent period of months. Considerable tissue sparing was observed in hippocampal and cortical regions after CHPG treatment, which was associated with significant improvements in both sensorimotor (beam walk) and cognitive (MWM) function.

Chronic neurological deficits are characteristic of moderate to severe TBI, although such deficits may stabilize or improve over time - likely reflecting endogenous plasticity. The DTI studies extend the observations from T2-weighted MRI by demonstrating significantly better preservation of white matter tracks in the brain at four months in the CHPG-treated TBI group as compared to controls. DTI detects directionality of water diffusion. After TBI, FA increases, whereas mean diffusivity, or the directed diffusion of water, typically along white matter tracks, is reduced [58,59]. These techniques have been used to reflect the integrity of white matter tracks (tractography) in the CNS [58]. FA has been shown to be negatively correlated with deficits in memory performance [59], consistent with our observations that CHPG treatment reduced this measure while improving cognitive performance.

## Conclusions

In summary, we demonstrate that markedly delayed treatment with CHPG one month after TBI significantly reduces subsequent lesion progression and white matter damage, with marked improvement in sensorimotor and cognitive function. Such delayed CHPG treatment attenuates neurodegeneration in the CA3 and dentate gyrus subfields of the hippocampus, areas that show significant neuronal loss after TBI and are associated with cognitive impairment after injury [35,60,61]. CHPG administration significantly attenuates the activation state of microglia in the injured cortex, although it did not alter the total number of microglia. The fact that co-administration of an mGluR5 antagonist blocked the protective effects of CHPG indicates that its therapeutic actions were specific and mediated by mGluR5 receptors, consistent with our previously published *in vitro *data [19,21].

This study significantly extends the currently accepted therapeutic window for neuroprotection after head injury. It also provides mechanistic experimental support for microglial-mediated chronic neurodegeneration after TBI and introduces a novel therapeutic approach for brain trauma. Given the therapeutic efficacy of delayed CHPG treatment, and its ability to modify chronic neuroinflammatory processes after traumatic injury, similar strategies might prove beneficial for other chronic neurodegenerative disorders that show a significant inflammatory component.

## Abbreviations

aDW: average diffusion-weighted; ANOVA: analysis of variance; CA: *Cornu Ammonis*; CCI: controlled cortical impact; CHPG: (*RS*)-2-chloro-5-hydroxyphenylglycine; CNS: central nervous system; DTI: Diffusion tensor imaging; FA: Fractional anisotropy; Iba-1: ionized calcium binding adaptor molecule 1; icv: intracerebroventricular; mGluR5: metabotropic glutamate receptor 5; MRI: magnetic resonance imaging; MTEP: 3-((2-Methyl-4-thiazolyl)ethynyl)pyridine; MWM: Morris water maze; NADPH: reduced nicotinamide adenine dinucleotide phosphate; NF: nuclear factor; NMR: nuclear resonance imaging; PBS: phosphate-buffered saline; SEM: standard error of the mean; siRNA: small interfering RNA; TBI: traumatic brain injury; TE: echo time; TR: repetition time.

## Competing interests

KRB, DJL and AIF are listed as inventors for a use patent from Georgetown University for mGluR5 agonists in the treatment of neuroinflammation.

## Authors' contributions

KRB carried out the TBI surgeries, mouse behavior and T2-weighted MRI. DJL performed MRI analysis. DJL and BAS carried out imaging and stereology studies. JZ carried out the *ex vivo *DTI. KRB, DJL and BAS designed and coordinated the study and wrote the manuscript. AIF conceived the study and wrote the manuscript. All authors read and approved the final manuscript.

## Author information

AIF is the David S. Brown Professor in Trauma, Professor of Anesthesia, Anatomy & Neurobiology, Neurosurgery and Neurology at the Center for Shock, Trauma & Anesthesiology Research.
